# Temperament and Character in Psychotic Depression Compared with Other Subcategories of Depression and Normal Controls

**DOI:** 10.1155/2011/730295

**Published:** 2011-11-22

**Authors:** Jaap G. Goekoop, Remco F. P. De Winter

**Affiliations:** ^1^Department of Psychiatry, Leiden University Medical Center, B1, Albinusdreef 2, P.O. Box 9600, 2300 RC Leiden, The Netherlands; ^2^Parnassia, Psycho-Medical Center, Monsterseweg 93, 2553 RJ The Hague, The Netherlands

## Abstract

*Background*. Support has been found for high harm avoidance as general vulnerability trait for depression and decreased self-directedness (SD) as central state-related personality change. Additional personality characteristics could be present in psychotic depression (PD). Increased noradrenergic activation in PD predicts the involvement of reward dependence (RD). *Methods*. The data during the acute episode and after full remission from the same subjects, that we used before, were reanalyzed. The dependence of the 7 dimensions of the Temperament and Character Inventory version 9 on PD, three other subcategories of depression, and a group of normal controls was tested by MANCOVA. *Results*. Low RD at both time points, and low Cooperativeness during the acute episode, were found as additional characteristics of PD. *Conclusion*. The combination of two premorbid temperaments, high HA and low RD, and the development of a state-related reduction of two character functions, SD and CO, may be the precondition for the development of combined depressive and psychotic psychopathology.

## 1. Introduction

The relation between personality and depression is complex, because of the many different types of relationships that have been found. Personality characteristics may be operative as premorbid vulnerability traits, pathoplastic traits, reversible state-dependent changes of personality that may have a pathoplastic or pathogenetic function, and even irreversible scars. Moreover, each subcategory of depression could have specific characteristics in one or more of these areas. The present study focuses on trait and state-related characteristics of personality in major depression with psychotic features, hereafter called psychotic depression (PD). As far as we know, this type of investigation has not been carried out before. This is a shortcoming not only from the theoretical perspective, but also from the practical point of view, since particularly vulnerability traits and pathogenetic personality changes could have important therapeutic consequences. 

The present study is part of a series of investigations in the same patient sample that aimed at the stepwise development of knowledge of subcategories of depression and of depressive disorders in general, by using (a) multidimensional description of the complex clinical pictures of subcategories of depression based on global dimensions of psychopathology [1] assessed by the comprehensive psychopathological rating scale (CPRS) [2], (b) multidimensional description of personality characteristics assessed by the Temperament and Character Inventory (TCI version 9) [3], (c) neurobiological characterization by means of plasma concentrations of arginine-vasopressin (AVP), cortisol and norepinephrine (NE), as well as their correlations, (c) family history of depression [4], and (d) a two-year followup. 

According to the TCI, personality can be conceived as a multidimensional construct comprising lower and higher levels of personal functioning and coping called temperament and character, respectively [3]. Whereas temperament involves automatic adaptation via conditioned response patterns, character is thought to involve conscious-adaptive information processing. The model comprises four temperament dimensions, called harm avoidance (HA), reward dependence (RD), novelty seeking (NS) and persistence (PER), and three character dimensions, called self-directedness (SD), cooperativeness (CO), and self-transcendence (ST). In the TCI version 9, HA consists of anticipatory worry versus optimism (HA1), fear of uncertainty versus confidence (HA2), shyness versus gregariousness (HA3), and fatigability and asthenia versus rigor (HA4). RD consists of sentimentality versus insensitivity (RD1), attachment versus detachment (RD3), and dependence versus independence (RD4). NS consists of exploratory excitability versus rigidity (NS1), impulsiveness versus reflection (NS2), extravagance versus reserve (NS3), and disorderliness versus regimentation (NS4), and PER of one facet called persistence versus irresoluteness. SD consists of acceptance of responsibility for one's own choices instead of blaming other people and circumstances (SD1), identification of individually valued goals and purposes versus lack of goal direction (SD2), development of skills and confidence in solving problems (resourcefulness) versus apathy (SD3), self-acceptance versus self-striving (SD4), and congruent second nature versus personal distrust (SD5). CO consists of social acceptance versus intolerance (CO1), empathy versus social disinterest (CO2), helpfulness versus unhelpfulness (CO3), compassion versus revengefulness (CO4), and pure-hearted principles versus self-advantage (CO5). ST comprises self-forgetful versus self-conscious experience (ST1), transpersonal identification (i.e., identification with nature) versus self-differentiation (ST2), and spiritual acceptance versus rational materialism (ST3). 

Our investigations started by the development of two new subcategories of depression in the field of melancholic or endogenous depression [5]. We first found support for a highly anxious-retarded (HAR) subcategory that was derived from the melancholic subtype according to the DSM-IV. Thereafter, we found support for a subcategory of depression with above-normal plasma arginine-vasopressin (ANA) concentration. At the level of personality characteristics, all patients with depression had increased harm avoidance (HA) after full remission compared with control subjects [6]. Fully remitted patients with HAR and ANA depression appeared to have in addition low self-directedness (SD) and low cooperativeness (CO), respectively, [6, 7]. Depression at large further appeared to be characterized by a state-related reduction of SD and increase of HA [6], while ANA depression in addition had a state-related reduction of reward dependence (RD) [7]. At the neurobiological level we found support for the HAR and ANA subcategories to have a genetic increase of the pituitary vasopressin receptor function and of the vasopressin synthesis, respectively, [5], while untreated depression at large eventually appeared also to have evidence of increased vasopressinergic function [8]. Recently we found the change of the character dimension of SD to be most directly related to the change of depressive symptoms [9]. This implies that rather than high HA, which we assume to be the most general vulnerability trait for depression, the state-related decrease of SD may represent the most general and central pathogenetic step for the actual development of depressive psychopathology. 

In the present study we searched for the additional personality characteristics of PD. Most recently we detected an increased concentration of plasma norepinephrine and evidence of increased noradrenergic-vasopressinergic activation in this subcategory [10]. Since measures of noradrenergic function have been associated with RD [11–16], we hypothesized PD to be specifically related with this dimension of temperament. 

In this study of PD we used three other subcategories of depression and a group of normative subjects as control groups. As a consequence of the stepwise procedure of our previous investigations, several subcategories that had been developed first appeared to have a small percentage of patients with features that eventually came out to be the defining characteristics of one or more other distinct subcategories. The increased AVP concentration initially found in HAR depression [17] appeared to belong to the defining characteristic of the ANA subcategory [18], and the psychotic features found in HAR as well as in ANA depression appeared to be better conceived to define a distinct subcategory of psychotic depression (PD) than as the phenotype of the severest form of HAR or ANA depression [10]. These results suggest that better delimitations between the subcategories of HAR and ANA depression as well as PD should be made by elimination of the initially accepted overlap. In this study we therefore used these better delimitated subcategories, and we tested whether the previously found relations with the HAR and ANA subcategories do still hold after the elimination of overlap. 

The data set of the same patient sample that we used before [6] was reanalyzed. We defined 4 subcategories of depression: PD, ANA depression without psychotic features (ANA-R), HAR depression without psychotic features and with normal vasopressin concentration (HAR-R), and the remaining group of all other depressed patients as the fourth subcategory. As before, we used the data of the personality characteristics at time of entrance in the study and after 2 years during full remission. The four depressive subgroups together with a control group of normative subjects were used as fixed factors in a MANCOVA, in which the 7 TCI version 9 dimensions served as the dependent variables, and age and gender as covariates. During the acute episode we hypothesized that HAR-R is not specifically related with any personality dimension [6], and that ANA-R is related with both reduced CO and RD [7]. After full remission, HAR-R was hypothesized to depend specifically on low SD [6], and ANA-R on low CO [7]. We hypothesized that PD is related to RD at least during the acute episode, in which we found the increased plasma NE concentration. The state-relatedness of RD and other dimensions of personality in PD was explored. Eventually, we explored the role of the subscales of the TCI version 9 dimensions that were found to be related to PD, by comparing all four subcategories of depression.

## 2. Methods

### 2.1. Patients and Control Subjects

We used the same sample of acutely depressed patients referred to our institute, that we used before in our investigation of the relation between TCI version 9 data and the ANA and HAR subcategories of depression [6, 7]. For the present study we selected the data from the start of the study (t1; *n* = 78) and after 2 years (t7; *n* = 58). The inclusion criteria were major depression (DSM-IV (APA, 2000)) and a score >20 on the Montgomery-Asberg rating scale (MADRS) [19]. All patients were recently referred to the psychiatric institute and referred to the study by the psychiatrist who made the initial diagnosis. After confirmation of the diagnosis by RFPdeW, using a semistandardized interview, the patient was asked to participate in the study. Exclusion criteria were bipolar disorder; treatment with lithium, carbamazepine, or valproate; first episode of major depression at age 60 years or older; alcohol or drug abuse or dependence; pregnancy; clinical evidence of a condition associated with abnormal plasma AVP release, such as the syndrome of inappropriate Secretion of antidiuretic hormone. The noncompleters of the study were just lost in the followup. Those lost and those remaining in the followup did not differ on clinical or demographic parameters (neuroticism, number of previous episodes, duration of current episode, family history of depression, psychotic depression, atypical depression, melancholia and anxious-retarded subtype, severity, and age, gender, and education) [20].

The MADRS was used as this scale has been developed from the CPRS and has maximal sensitivity to change in depressed patients. The use of the score >20 was derived from the general rule of thumb at the time of the study to take 1/3 of the maximal scale score for the delimitation of sufficient severity. The MADRS was preferred above the Hamilton rating scale for depression (HAMD) since the MADRS has high unidimensionality [21], and the (HAMD) an insufficiently reproducible factor-structure [22]. The MADRS was preferred above the Beck depression inventory, since the MADRS is less influenced by maladaptive personality traits [23]. 

Psychotic depression (PD; *n* = 10) was diagnosed according to the DSM-IV-criteria [24]; ANA-R (*n* = 13) was defined by a concentration of plasma AVP > 5.6 pg/mL and [18] and the absence of PD; HAR-R (*n* = 14) was defined by the combination of anxiety and retardation scores ≥ the median [25] and the absence of ANA and PD. The remaining group of all other depressed patients was the fourth depressive subcategory (all other depressed; *n* = 41). After two years of followup (t7), 41 of the 58 completers of the followup were in full remission (71%) defined as no more than 2 items of the DSM-IV criteria for major depressive episode during two weeks corresponding with the criteria proposed by Frank et al. [26]. In a previous study using somewhat more rigid criteria, we found 65% to have full remission [27]. In the present study, the DSM-IV criteria for major depression were assessed by using the corresponding CPRS items. The CPRS items were rated from 0 to 6, covering the two weeks preceding the assessment. A score >2 was taken to represent the DSM-IV severity criterion of a symptom being more present than absent. Increased appetite and weight were rated separately. The number of patients in each subcategory at t7 was PD: *n* = 6; ANA-R: *n* = 9; HAR-R: *n* = 8; all other depressed patients: *n* = 18. 

Normal control subjects (*n* = 86) were selected from a normative sample [28] that was recruited at random from the national telephone book, as described in the study of the relation between the HAR subcategory and personality [6]. For reason of comparability with our previous studies, the number of control subjects was kept the same. The differences between the scores of these 86 normal controls and the scores of the normative sample by Cloninger et al., [3] (higher HA, lower PER, and lower ST scores compared to Cloninger's normative sample (HA: 15.4 (7.1) versus 12.6 (6.8), PER: 4.4 (1.9) versus 5.6 (1.9), ST: 11.2 (6.1) versus (19.2 (6.3)), correspond with the data of the full sample of the Dutch validation study [28] and seem to be due to cultural differences. The controls were not investigated for anxiety or depression.

### 2.2. Personality

In patients and controls we used the scores of the three character dimensions of self-directedness (SD), cooperativeness (CO) and self-transcendence (ST), and the four temperament dimensions of harm avoidance (HA), reward dependence (RD), novelty seeking (NS), and persistence (PER) of the TCI version 9 [3, 28]. Scores of the subscales of these main dimensions were only available in the patient sample.

### 2.3. Statistical Analysis

The four depressive subgroups (PD, ANA-R, HAR-R, and all other depressed patients) were used together with a control group of normative subjects, as fixed factors in a MANCOVA, in which the seven TCI version 9 scores served as the dependent variables, and age and gender as covariates. For the test of the dependence of PD on RD, we used a *P* value of 0.05. To correct for chance effects in case of additional relations between PD and any of the 7 TCI measures we used a *P* value of 0.007 in the exploratory analyses. The relations between HAR-R and SD and between ANA-R and CO, that we previously found in the same sample for the HAR and ANA subcategories, were tested by using a *P* value of 0.05. The one sample binomial test and one-sample Kolmogorov-Smirnov tests were used to test the homogeneity of the distribution of gender and age, and RD and CO at start and at the end of the followup. The analyses were carried out with the SPSS version 18.0.

## 3. Results 

### 3.1. Personality Characteristics during the Acute Depressive Episode

Table 1 shows the scores on the 7 TCI version 9 scales at the start of the study, when all patients fulfilled the criteria for major depressive episode. Corresponding with our previous analysis in the same patients sample [6], a MANCOVA with the whole depressed group and the control group as fixed factors, showed that depression (*n* = 78) had significantly increased HA (*F* = 76.625; *P* < 0.001) and decreased SD (*F* = 92.755; *P* < 0.001), while CO and RD were nonsignificantly reduced (*F* = 5.118; *P* = 0.025, and *F* = 5.885; *P* = 0.016, resp.). A MANCOVA that used the 4 subcategories of depression and the control group as 5 fixed factors, showed that PD was characterized by low CO (*t* = −2.949; *P* = 0.004) and low RD (*t* = −2.717; *P* = 0.007), and ANA-R similarly still also had low RD (*t* = −2.718; *P* = 0.007) with low CO (*t* = −2.237; *P* = 0.027). All 4 subcategories had high HA (*t* = 4.131, 4.504, 5.804, and 7.945; *P* < 0.001) and low SD (*t* = −4.020, −4.136, −5.493, and −6.976; *P* < 0.001). The high HA and low SD were most strongly present in the group of all other depressed patients. 

A similar analysis within the sample of depressed patients showed that PD was characterized by a statistically nonsignificant low score on the dependence subscale of the RD dimension in comparison with the subcategory of all other depressed patients (*t =* −1.857; *P* = 0.067), and low scores on the compassion versus revengefulness (*t =* −2.004; *P* = 0.049) and (nonsignificantly) pure-hearted versus Selfserving subscales (*t =* −1.822; *P* = 0.072) of the CO dimension, while the ANA-R subcategory was characterized only by low compassion versus revengefulness (*t =* −2.018; *P* < 0.047) from the CO dimension. Given the fact that 3 subscales could be involved in low RD and 5 subscales in low CO, these relations were not statistically significant after correction for multiple assessment.

### 3.2. Personality Characteristics after Full Remission of the Depressive Episode 

In the whole group of fully remitted patients (see Table 2) MANCOVA showed that HA was high (*t =* 4.645; *P* < 0.001) and ST low (*t =* −2.008; *P* = 0.047) compared with the control subjects. A MANCOVA that used the 4 subcategories of depression and the control group as 5 fixed factors, showed that PD (*n* = 6) was only characterized by low RD (*t =* −2.236; *P* = 0.027), while the ANA-R subcategory (*n* = 9) had only low CO (*t =* −2.761; *P* = 0.007). The HAR-R subcategory (*n* = 8) had low SD (*t =* −2.001; *P* = 0.048). The ANA-R and HAR-R subcategories and the group of all other depressed patients (*n* = 18) had significantly high HA (*t =* 1.981; *P* = 0.050, *t =* 2.770; *P* = 0.007, and *t =* 3.186; *P* = 0.002, resp.), while the PD subcategory had a similarly increased HA combined with a low standard deviation, that nonetheless just lacked statistical significance (*P* = 0.053). A comparison of the data from Tables 1 and 2 shows that CO in PD and RD in the ANA-R subcategory showed some state-related increase resulting in the absence of a difference compared with the normal control subjects, while RD in PD and CO in ANA-R had not changed very much. These data suggest state-dependent reductions of CO in PD and of RD in ANA-R. 

A similar analysis, within the sample of fully remitted patients, showed that PD was characterized by a low score on the attachment subscale of the RD dimension in comparison with the subcategory of all other depressed patients (*t =* −2.433; *P* = 0.020), and a low score on the Compassion versus revengefulness subscale of the CO dimension (*t =* −2.309; *P* = 0.027), while the ANA-R subcategory was characterized by low compassion versus revengefulness (*t =* −4.538; *P* < 0.001) and a low score on the pure-hearted versus selfserving subscale (*t =* −3.631; *P* = 0.001) of the CO dimension, combined with a low Dependence (*t =* −2.153; *P* = 0.038) within the RD dimension. After correction for multiple assessment only the relations between the two subscales of CO and ANA-R remained statistically significant. 

 Finally, the one-sample binomial test and one-sample Kolmogorow-Smirnov tests showed that in the subcategory with PD the null hypothesis of the distribution of gender and age, and RD and CO at start and at the end of the followup should be retained (*P* = 0.508; *P* = 0.666; *P* = 0.982; *P* = 0.993; *P* = 0.982; *P* = 0.998, resp.). This supports the relative stability of the PD sample.

## 4. Discussion

### 4.1. Personality Characteristics of PD and the General Characteristics of Depressive Disorders

This study confirmed the hypothesis that PD is related to RD. We found that RD was low compared with normal control subjects both during the acute episode and after full remission. This supports that low RD is an additional vulnerability trait for PD. The present study unexpectedly also showed that PD is related with a probably state-dependent reduction of CO. As far as the most general characteristics of depression are concerned, we found that the high HA and state-related reduction of SD found in the whole group of depressed patients [6] were present in all four subcategories (PD, ANA-R, HAR-R and all other depressed patients). The high HA in PD after full remission just lacked statistical significance. This is probably due to the low number of fully remitted PD patients, as the HA score had the same high level combined with even a relatively low standard deviation compared with the other subcategories of depression. Despite this low number of PD patients, the combined data strongly suggest that PD is characterized by two premorbid temperamental traits, namely, high HA and low RD, and two state-related changes of character function, involving SD and CO. The nonpsychotic subcategories on the other hand appeared to be characterized only by high HA and state-related reduction of SD. Since HA and SD are generally negatively correlated and RD and CO generally positively [3, 28–39], and these dimensions may therefore be the temperament and character aspects of more global domains of personality involved in vulnerability to stress (or resilience) and affiliative behavior, the psychotic depressive disorder could be seen as having paired vulnerability traits and state-related changes in both of these global domains of personality in contrast to the nonpsychotic depressive disorders. The statistically nonsignificant relations found within the group of depressed patients between subscales of RD and CO suggest that the attachment subscale of the RD dimension could be involved as a trait characteristic in PD. low compassion versus revengefulness could be involved PD as a state-related characteristic, but possibly also as a trait characteristic. Reanalysis with the improved subscales of the TCI-R and a larger sample of PD patients will be necessary to get a better insight into the role of specific subscales of RD and CO in PD. 

Although low RD may result in insufficient automatic affinity with the feelings of other people, and reduced CO in insufficient appraisal of their opinion, which could contribute to insufficient correction of delusional thoughts, the two characteristics could also have no causal meaning, as there may be unexplained variance due to pathogenetic variables not included in the analysis.

### 4.2. The Effect of Elimination of Overlap between PD, HAR, and ANA Depression

The present study further showed that the elimination of overlap between the PD, ANA, and HAR subcategories did not substantially affect our previous findings of specific low character scores of SD and CO after full remission in HAR and ANA depression, and the state-related change of RD in ANA depression. In our previous study ANA-depression had reduced RD (*F =* 8.466; *P* = 0.004) and reduced CO (*F =* 8.052; *P* = 0.006) during the acute episode compared with the control subjects [7]. The fact that in the present study the same relations with ANA-R were less strong than in the nonrevised ANA subcategory is presumably due to the lower number of subjects, as the mean values during the acute episode did not differ much (RD = 12.8; sd = 4.3 versus 12.8; sd = 4.8, and CO = 29.2; sd = 5.6 versus CO = 29.4; sd = 6.1, resp.). The mean of the CO score in the ANA-R subcategory after full remission (CO = 28.4; sd = 3.3) was nearly identical to that of ANA depression (CO = 28.5; sd = 3.4), and the strength of the relation was only weakly reduced (*P* = 0.007) compared to before the elimination of overlap (*P* = 0.003) [7]. This may be due to the fact that only two psychotic patients were eliminated from the group of fully remitted patients with ANA depression. In the group of HAR-R patients in full remission we found a mean SD score of 27.3 (sd = 7.6) and a *P* value of this relation of 0.048. This mean SD value was somewhat lower than that found in unrevised HAR depression (SD = 28.4; sd = 7.9) [6], which suggests that the elimination of overlap has resulted in a subcategory with increased validity. 

### 4.3. Noradrenergic Mechanism Involved in the Low RD and Decreased CO in PD

As far as we know this is the first report on temperament and character in PD. The finding of the low score on the temperament dimension of RD confirmed the hypothesis that a noradrenergic mechanism is involved in PD. This hypothesis was derived from earlier findings of relations between RD and noradrenergic function [11–16]. The fact that RD was decreased in this psychotically depressed subcategory, while the plasma NE concentration was increased (*F =* 4.993; *P* = 0.023) [10], could be seen as contrasting with the findings of a positive correlation between RD and concentrations of the main metabolite of NE in urine [15, 16]. However, as the NE concentrations in PD are for a large part above the normal reference value [10], a concentration-dependent inverted U-curve relationship between NE release and the function of the central target regions involved in the affiliative behavior of RD could be involved. As the personality characteristics of PD and ANA-R seem to form mirror images, as far as the RD and CO traits and state-related changes of CO and RD are concerned, the opposite neurobiological mechanisms that we found in PD and ANA-R, namely, a positive NE-AVP correlation in PD [10] and a negative AVP-NE correlation in ANA-R [40], could be involved. 

These data further support the usefulness of the original concept of the TCI as a scale that corresponds with the sociobiological origins of personal differences. Next to the above mentioned findings in the field of the noradrenergic system, the model is supported by relations between the serotonergic system and both HA and SD [27], as well as the dopaminergic system and NS [20].

### 4.4. Comparison between PD and Schizophrenic Disorders

The personality characteristics of high HA and low RD after full remission in PD and state-dependent reductions of the character dimensions of SD and CO, that we found to differentiate PD from other depressive disorders, appear to have some correspondence with the personality characteristics found in schizophrenic patients [41]. The patients in the latter study also had the combination of high HA and low RD, as well as the combination of low SD and low CO. The main differences compared with the PD patients of the present study was an additional high ST in the schizophrenic patients and the probable reversibility of the decreases of SD and CO. The comparison with the results of the present study suggest that the combination of high HA and low RD may constitute a general vulnerability configuration for psychotic disorders, and that the reduced character dimensions of SD and CO may function as the chronic or reversible character deficits that are involved in the actual development of the psychotic dysregulation. 

Limitations of the study are the lack of subscale scores of the TCI version 9 in the normal control subjects, as well as the small number of patients with PD. The support for a specific vulnerability trait for the psychotic component of PD may imply that drugs that enhance affiliative behavior could be useful, and that specific psychotherapeutic attention could be directed towards the improvement of this type of behavior.

## Figures and Tables

**Table 1:** Temperament and character (standard deviation between brackets) during the acute depressive episode of psychotic depression (PD), ANA-R depression, HAR-R depression, and all other depressed patients, as well as in healthy controls (***P < 0.001; **P < 0.0071; *P < 0.05: *P* values compared with controls).

	*n*	HA	RD	NS	PER	SD	CO	ST
Depressive episode	78	25.4*** (6.1)	14.7 (3.9)	17.7 (6.6)	4.7 (1.9)	23.1*** (7.1)	31.4 (6.1)	10.6 (6.0)
PD	10	24.7*** (7.8)	12.7** (3.1)	18.9 (7.1)	4.2 (1.2)	23.4*** (6.9)	28.1** (6.7)	13.8 (5.8)
ANA-R	13	24.1*** (6.8)	12.8* (4.8)	16.3 (6.4)	5.5 (1.8)	24.1*** (8.4)	29.4* (6.0)	10.1 (5.4)
HAR-R	14	26.4*** (5.9)	16.1 (3.2)	16.4 (4.1)	4.9 (2.4)	21.7*** (7.0)	32.6 (6.0)	12.3 (6.9)
All other depressed	41	25.7*** (5.7)	15.2 (3.8)	18.3 (7.2)	4.6 (1.9)	23.3*** (6.9)	32.5 (5.7)	9.4 (5.6)
Healthy controls	86	15.4 (7.1)	16.1 (4.1)	19.5 (5.5)	4.4 (1.9)	32.3 (6.4)	33.3 (5.2)	11.2 (6.1)

**Table 2:** Temperament and character (standard deviation between brackets) after full remission in psychotic depression (PD), ANA-R depression, HAR-R depression, and all other depressed patients, as well as in healthy controls (***P < 0.001; **P < 0.0071; *P < 0.05: compared with controls).

	*n*	HA	RD	NS	PER	SD	CO	ST
All remitted patients	41	21.7*** (7.7)	15.5 (3.6)	20.0 (6.5)	4.6 (1.9)	29.8 (7.7)	32.9 (6.0)	8.8* (6.3)
PD	6	22.2 (5.8)	12.8* (4.5)	20.3 (7.3)	3.8 (1.3)	31.0 (5.4)	30.8 (8.4)	9.8 (3.8)
ANA-R	9	20.4* (9.3)	14.3 (4.2)	19.7 (5.5)	4.9 (1.5)	30.2 (7.8)	28.4** (3.3)	8.3 (4.3)
HAR-R	8	22.3** (9.0)	16.1 (3.6)	19.4 (7.6)	4.5 (1.9)	27.3* (7.6)	32.6 (6.6)	9.9 (7.7)
All other depressed	18	21.9** (7.4)	16.7 (2.3)	20.4 (6.8)	4.7 (2.2)	30.3 (8.7)	35.9 (4.2)	8.1 (7.3)
Healthy controls	86	15.4 (7.1)	16.1 (4.1)	19.5 (5.5)	4.4 (1.9)	32.3 (6.4)	33.3 (5.2)	11.2 (6.1)
